# Similar PAH Fate in Anaerobic Digesters Inoculated with Three Microbial Communities Accumulating Either Volatile Fatty Acids or Methane

**DOI:** 10.1371/journal.pone.0125552

**Published:** 2015-04-15

**Authors:** Florence Braun, Jérôme Hamelin, Anaïs Bonnafous, Nadine Delgenès, Jean-Philippe Steyer, Dominique Patureau

**Affiliations:** 1 INRA, UR0050, Laboratoire de Biotechnologie de l’Environnement, Avenue des Etangs, Narbonne, F-11100, France; 2 ADEME, French Environment and Energy Management Agency, 20 avenue du Grésillé-BP 90406, F-49004, Angers, Cedex 01, France; University of Kansas, UNITED STATES

## Abstract

Urban sludge produced on wastewater treatment plants are often contaminated by organic pollutants such as polycyclic aromatic hydrocarbons (PAH). Their removal under methanogenic conditions was already reported, but the factors influencing this removal remain unclear. Here, we determined the influence of microbial communities on PAH removal under controlled physico-chemical conditions. Twelve mesophilic anaerobic digesters were inoculated with three microbial communities extracted from ecosystems with contrasting pollution histories: a PAH contaminated soil, a PCB contaminated sediment and a low contaminated anaerobic sludge. These anaerobic digesters were operated during 100 days in continuous mode. A sterilised activated sludge, spiked with 13 PAH at concentrations usually encountered in full-scale wastewater treatment plants, was used as substrate. The dry matter and volatile solid degradation, the biogas production rate and composition, the volatile fatty acids (VFA) production and the PAH removals were monitored. Bacterial and archaeal communities were compared in abundance (qPCR), in community structure (SSCP fingerprinting) and in dominant microbial species (454-pyrosequencing). The bioreactors inoculated with the community extracted from low contaminated anaerobic sludge showed the greater methane production. The PAH removals ranged from 10 % to 30 %, respectively, for high and low molecular weight PAH, whatever the inoculums tested, and were highly correlated with the dry matter and volatile solid removals. The microbial community structure and diversity differed with the inoculum source; this difference was maintained after the 100 days of digestion. However, the PAH removal was not correlated to these diverse structures and diversities. We hence obtained three functional stable consortia with two contrasted metabolic activities, and three different pictures of microbial diversity, but similar PAH and matter removals. These results confirm that PAH removal depends on the molecule type and on the solid matter removal. But, as PAH elimination is similar whether the solid substrate is degraded into VFA or into methane, it seems that the fermentative communities are responsible for their elimination.

## Introduction

Due to both domestic and industrial human practices, organic micropollutants like polycyclic aromatic hydrocarbons (PAH) are detected at various concentrations in many environments like soils [1,2], sediments [3–5], waters [6] and biota [7]. They are produced by partial combustion of organic matter during fossil fuel or wood combustion, waste incineration, use of coal and petroleum refining and found in coal tar, crude oil, creosote, and roofing tar, or produced by dyes, plastics, and pesticides manufacturing. Considered as persistent and bioaccumulative compounds, they are also classified as endocrine disrupters by various environmental agencies.

Wasted domestic, industrial and runoff waters converge to the water treatment plant, where PAH accumulate in the sludge phase [8], due to their hydrophobic properties. In Europe, theses sewage sludge are mainly used as fertilizers but need to be previously stabilized. Different processes are used for stabilization, like anaerobic digestion, leading to the degradation of organic matter into biogas (CO_2_ and CH_4_).

Anaerobic digestion consists of four stages where various microorganisms are involved: the hydrolysis, the acidogenesis, the acetogenesis and the methanogenesis. PAH biodegradation was already reported under methanogenic conditions [9,10], likewise under other anoxic conditions, i.e. sulfate-reducing or denitrifying conditions [11–13]. Previously, Christensen *et al*. [14] showed that the degradation of low molecular weight PAH as naphthalene was possible if hydrogenotrophic *Archaea* are available to remove H_2_ produced by oxidation of the PAH. Recently, a thermodynamic landscape study demonstrated the exergonic character of PAH biodegradation under methanogenic condition: they indicated that PAH may be converted to acetate and H_2_, these latter being transformed to methane either through acetoclastic methanogenesis or syntrophic acetate oxidation and CO_2_ reduction [15]. The observations of Chang *et al*. [16] with an inhibitor of methanogenesis that partially hindered PAH degradation with a complete elimination of methanogenic *Archaea* and some *Bacteria* also suggested that degradation could be associated with a previous step of organic matter anaerobic digestion (i.e acidogenesis or acetogenesis). However, little is known on the microbial communities implied in the PAH degradation of complex and mixed culture (not enriched culture). Indeed, different microorganisms have been already identified but it was not clear whether all those species were implicated in the first step of degradation or if they were degrading by-products or contributing to the global electrons mass balance. Identified species are: (i) an hydrogen utilizing Archaea belonging to the *Methanobacteriaceae* family [14], (ii) bacterial clones related to the genera *Cytophaga* and *Acholeplasma*, (iii) an archaeal clone closely related to the order *Methanosarcinales* [17], (iv) an Archaea related to the genus *Methanosarcina*, (v) bacteria related to *Spirochaetes* and *Firmicutes* [16], (vi) a bacterium belonging to the *Clostridium* genus which anaerobically metabolized the acenaphtalene and fluorene but degraded partially the phenanthrene, anthracene and pyrene [18], and (vii) archaeal members closely affiliated with *Methanosaeta* and *Methanoculleus* and bacterial members closely related to *Clostridiaceae* [19]. Only one paper from Zhang et al. [20] refers to the use of Single Isotope Probing and have identified anthracene degraders within the genera *Methylibium* and *Legionella*, while another species was an unclassified *Rhizobiales*.

Some authors stipulate that the potential of PAH degradation relies on the sludge type [21]: PAH removal can double according to some sludge characteristics, like the fraction of readily biodegradable particles or the concentration of dissolved and colloidal matter. Indeed, the sludge composition has an impact on either the PAH bioavailability (interaction between organic matter and pollutants) or the expression of the biodegradation potential of the PAH through cometabolism (co-consumption of PAH and organic matter for growth) or both. Up to now, the experiments conducted under such conditions are unable to disentangle which impact is the most reliable to optimize the system. The relative importance of these limiting factors on PAH removals is indeed difficult to assess. Disentangle compounds availability from biodegradation potential while better understanding the microbial key players, is the challenge of this study.

To analyze the role of a microbial community on one process, avoiding the effect of different organo-mineral composition of its natural environment, it is a current practice in applied ecological studies to extract the entire microbial communities from their environment and to re-seed them into microcosms under the same controlled conditions [22]. We applied this ecological strategy of transplantation (common garden experiment) to study the influence of the microbial communities extracted from various environments on PAH removals. With this strategy, the extracted cells can be studied under the same condition without being disturbed by their native environment.

The aim of this study was thus to determine the role of microbial communities on the anaerobic fate of 13 PAH present in a single and well defined sludge over the time. For this, the entire microbial communities originating from three environments (soil, sediment and sludge) with contrasting pollution histories were extracted with a new developed protocol [23] and resettled into three series of anaerobic mesophilic reactors filled with a defined, spiked and sterilized sludge. The reactors were run during 100 days. First, to characterize the reactors performances, the PAH removals, the production of biogas and the other operational parameters (dry matter and volatile solids, volatile fatty acids concentration, chemical oxygen demand reduction) were collected. Second, bacterial and archaeal communities characterization by fingerprinting and high-throughput sequencing were related to changes in functioning by Principal Component Analysis (PCA). This is the first report in which a strategy of community transplantation has been applied to investigate and to characterize the microbial potential of anaerobic PAH removal under the same well-controlled environmental conditions.

## Materials and Methods

### Chemicals

Thirteen PAH (S1 Table) were mixed to spike the sludge used as reactor feed. The 13 PAH used (fluorene, phenanthrene, anthracene, fluoranthene, pyrene, benzo(a)anthracene, chrysene, benzo(b)fluoranthene, benzo(k)fluoranthene, benzo(a)pyrene dibenzo(a,h)anthracene, benzo(g,h,i)perylene and indeno(1,2,3,c,d)pyrene) were chosen among the molecules identified as priority chemical by US-EPA. All solvents were purchased from J.T. Baker. PAH powders were obtained from Dr Ehrenstorfer GmbH. Each compound was dissolved in dichloromethane at 2 g.L^-1^. A spiking mix was then prepared in acetonitrile at 100 mg.L^-1^ for each PAH except for indeno(1,2,3,c,d)pyrene (20 mg.L^-1^). The standard solution of PAH in acetonitrile at 10 mg.L^-1^ was provided by Dr Ehrenstorfer GmbH. For quantification, the standard solutions were diluted to obtain 6 calibration levels from 10 to 1,000 μg.L^-1^. Standards were stored at -20°C.

### Reactor inocula and reactor feed preparation

#### Microbial communities used as inoculum

Microbial cells were extracted from three ecosystems with contrasting pollution histories: 10 g of a PAH contaminated soil (eco1), 10 g of a PCB contaminated sediment (eco2) and 10 g of dry matter of a low contaminated anaerobic sludge (eco3). The PAH contaminated soil was sampled at a coal factory in the north of France, the PCB contaminated sediment came from a French industrial valley and the sludge from an urban wastewater treatment plant located in an industrial site with a long—term micropollutant contamination history.

#### Extraction of microbial cells used as inoculum

The cell extraction was performed with a cell flotation Gentodenz treatment. Extraction by cell flotation is efficient for soil and sediment samples but for sludge samples, an enzymatic treatment is necessary to recover viable microbial cells [23].

To obtain the cells extract from sludge, a volume corresponding to 10 g of dry matter was centrifuged (15,000 g, 20 min). The resulted pellet was suspended in 250 mL of 80 mM phosphate buffer with MgCl_2_ and sodium triphosphate pentabasic (STTP, Sigma) at final concentration of 20 mM and 50 mM, respectively. The enzymatic pre-treatment consisted of a mixture of α-amylase, pectinase, cellulose and DNase as used by Braun *et al*. [23]. The cell suspension was washed by centrifugation at 15,000 g for 20 min at 4°C and the resulted pellet suspended in 250 mL of physiological water. The suspension was centrifuged at 15,000 g for 20 min at 4°C and the whole pellet was suspended into 50 mL of Polyvinylpolypyrrolidone (PVPP) buffer (130 mM NaCl, 7 mM Na_2_ HPO_4_, 3 mM NaH_2_PO_4_, 5 mM Na_2_ EDTA, pH 7.3; PVPP at concentration of 3.5 mg.mL^-1^; and 1% Tween 20). For the sample of soil and sediment, 10 g of sample were suspended in 50 mL PVPP buffer. For all sample, the suspension obtained was mixed in a blender (T15 basic; Ultra Turax) at maximum speed for 90 s in presence of ice and then agitated at 37°C overnight. To separate the cells from the other compounds of the samples, a high-speed centrifugation procedure with a density gradient elaborated with Gentodenz at a density of 1.3 g.mL^-1^ was used as described by Braun *et al*., [23].

The number of viable recovered bacterial cells used as inocula was estimated by quantitative PCR (q-PCR) and averaged 4.08 × 10^11^ bacteria.g^-1^_VS_ (varied from 1.90 × 10^11^ to 6.26 × 10^11^ *Bacteria*.g^-1^_VS_). The number of recovered archaeal cells of *eco1* was below the limit of quantification by q-PCR (8.6 × 10^6^ *Archaea*.g^-1^_VS_). In *eco2* and *eco3*, we estimated an average of 1.14 × 10^7^ and 9.25 × 10^10^ *Archaea*.g^-1^_VS_, respectively.

#### Preparation of the sludge to feed the reactor

An activated sludge sampled in an urban wastewater treatment plant (250000 person equivalent) was diluted in tap water to reach a concentration of 20 g_COD_.L^-1^. Its composition after dilution was the following: 16.3 g.L^-1^ Dry Matter (DM), 12.9 g.L^-1^ Volatile Solid (VS). Half of this sludge was spiked with the pollutant mix solution at a concentration of 5 μg.g_DM_^-1^ for each PAH except for indeno(1,2,3,c,d) pyrene (1 μg.g_DM_^-1^). The two sets of sludge, spiked or not, were distributed in 1 L bottles, respectively, that were sterilized at 120°C for 30 min and stored at -20°C. Each week, one bottle of each set of sludge was thawed overnight at 4°C before being used as reactor feed. Before use, the pH was adjusted at 7 with a sterilized NaCO_3_^-^ solution at 52 g.L^-1^. Samples were also taken from these feeding bottles for chemical and pollutant analysis. The sludge samples were never stored more than a week after thawing and were always manipulated under a laminar flow hood to guaranty the sterility of the feeding sludge.

### Experimental setup

For the start-up, 100 mL of the sterilized spiked sludge (at 1 g_DCO_.L^-1^) were inoculated with each extracted microbial community (*eco1*, *eco2* and *eco3*). Four reactors per microbial community were first conducted in fed-batch mode (about 100 days, until reaching 400 mL of reaction volume) to allow a gradual increase of the applied load and a progressive adaptation of communities to the experimental conditions. One of the 4 reactors was fed with the sterilized and non-spiked sludge as negative control and the 3 others with the sterilized and spiked sludge.

Then, all the 400 mL anaerobic reactors were fed in a continuous mode. The reactors were magnetically stirred and maintained under usual mesophilic temperature (35°C) in a culture chamber. Once a day, the pressure generated by the biogas production was recorded and the head space was adjusted at atmospheric pressure. Then, 20 mL of liquid was removed and collected in tanks at 4°C during 10 days before chemical and molecular analyses. The 20 mL of digested sludge were replaced in reactors by the same volume of fresh sludge. All these steps were carried out under sterile conditions. The composition of biogas was measured three times a week [24]. The 12 continuous reactors were thus operated at a fixed hydraulic retention time (HRT) of 20 days and an organic load of 1 g_COD_.L^-1^.day^-1^. The reactors were run during 5 HRT.

### Bioreactor performances

#### Chemicals analysis

The biogas production was measured using differences in pressure. The biogas composition (CH_4_, CO_2_, H_2_, and N_2_) was analyzed by gas chromatography (GC-14A, Shimadzu) [24]. The biogas production performances were normalized by degraded organic matter expressed as COD (mL.g_COD degraded_^-1^).

The dry matter (DM, g.L^-1^) was measured by weighing the sample after heating at 105°C during 24 h. Then, the volatile solids (VS, g.L^-1^) were measured by weighing the sample after heating at 550°C during 2 h. These parameters were used to evaluate the reactor performances such as the degradation rate of organic matter and to define the steady state of reactors.

The aqueous phase containing the dissolved and colloidal matter (DCM) was separated from total sludge by centrifugation (10,000 g, 20 min), followed by a filtration at 1.2 μm (GF/C filter, Whatman). The chemical oxygen demands (COD) in the total sludge (COD_tot_, g_O2_.L^-1^) and in the DCM phase (COD_DCM_, g_O2_.L^-1^) were determined thanks to Merck Spectroquant kits. The volatile fatty acids (VFA) [acetate (C2), propionate (C3), iso-butyrate (IC4), butyrate (C4), iso-valerate (IC5) and valerate (C5)] were determined in the aqueous phase by gas chromatography (GC800, Fisons Instruments). The concentration of VFA and the proportions of each VFA defined the fermentative or methanogenic activity of reactors.

Every 10 days, the accelerated solvent extraction of PAH was performed, on duplicate, on freeze-dried samples of the feeding and outlet total sludge according to Trably *et al*. [25]. Each extract was quantified in duplicates by high-performance liquid chromatography and fluorimetric detection [25]. The limit of detection was 20 ng/g_DM_ for each PAH. The PAH were grouped according to their molecular weight (MW). The PAH with a MW ≤ 200 g.mol^-1^ (fluorene, phenanthrene, anthracene) were qualified as low molecular weight PAH (LMW-PAH), those with 200 < MW ≤ 250 g.mol^-1^ (fluoranthene, pyrene, benzo(a)anthracene, chrysene) as medium molecular weight PAH (MMW-PAH) and those with MW > 250 g.mol^-1^ (benzo(b)fluoranthene, benzo(k)fluoranthene, benzo(a)pyrene dibenzo(a,h)anthracene, benzo(g,h,i)perylene and indeno(1,2,3,c,d)pyrene) as high molecular weight PAH (HMW-PAH).

#### Removal rate calculation

The comparison of the feed and outlet reactors parameters determines the removal efficiency of DM, VS or micropollutants. For the DM and VS, the average removal efficiencies were calculated at steady state on the last three ½ HRT (3 values). For each PAH, at each ½ HRT, we calculated an average removal efficiency with a minimum of 12 values by inoculum (4 values by reactor, each inlet and outlet sample being solvent extracted two times and analyzed once). At steady state, we calculated an average removal efficiency on the last three ½ HRT, so with a minimum of 36 values.

### Molecular analyses

The microbial communities were analyzed early in the experiment (HRT 1) and for the three last HRTs (HRT 3, 4 and 5). The structure of the *Bacteria* and *Archaea* communities was analyzed after DNA extraction, PCR amplification and separation by Capillary Electrophoresis-Single Strand Conformation Polymorphism (CE-SSCP). Quantitative PCR (Q-PCR) was used to quantify the number of *Bacteria* and *Archaea*.

#### DNA extraction

One mL of sludge sample was first centrifuged for 10 min at 10,000 g and the pellet was resuspended by vortexing in 385 μL of 4 M guanidine thiocyanate-tris-HCl pH 7.5 0.1 M and 115 μL of 10% (w/v) N-lauroyl-sarcosine (N-LS). The homogenous sample (500 μL) was stored at -20°C before DNA extraction according to the procedure described by Godon et al. [26]. The DNA concentrations were quantified by spectrophotometry using the Infinite 200 PRO NanoQuant (Tecan, France).

#### Polymerase chain reaction (PCR) amplification

To analyze the structure of the bacterial and archaeal communities, the V3 region of the 16S rRNA gene was amplified. The primers W49F and W104R were used for the Bacteria [27] and W274R and W275F for the Archaea [23] (S2 Table).

For the amplification of bacterial sequences, the PCR mixture contained 5 μL of 10X *Pfu* Turbo buffer, 200 nM deoxynucleoside triphosphates (dNTPs), 400 nM of each primer, 1.25 U of *Pfu* Turbo DNA polymerase (Stratagene, La Jolla, CA), 1 μL of genomic DNA, and water added to obtain a final volume of 50 μL.

For the amplification of archaeal sequences, the PCR mixture contained 2.5 μL of 10X *Pfu* Turbo buffer, 200 nM dNTPs, 500 nM of each primer, 0.625 U of *Pfu* Turbo DNA polymerase (Stratagene, La Jolla, CA), 0.5 μL of genomic DNA, and water added to obtain a final volume of 25 μL.

The PCR conditions for amplification of bacterial and archaeal sequences are presented in S3 Table. All reactions were stopped by cooling the mixture down to 4°C.

#### Capillary Electrophoresis-Single Strand Conformation Polymorphism (CE-SSCP)

The resulting PCR products were then separated by CE-SSCP that allows the separation of DNA fragments of the same size but with a different composition [28]. One μL of diluted PCR products was added to 18 μL of formamide and 1 μL of internal size standard Rox 400 HD (Applied Biosystems, California, USA) diluted 10 times. Each sample was then denatured (5 min at 94°C) and placed directly on ice for 5 min. CE-SSCP was performed using an ABI 3130 genetic analyzer (Applied Biosystems) equipped with four 50 cm capillary tubes filled with 5.6% of conformation analysis polymer (Applied Biosystems) in the corresponding buffer and 10% glycerol. The injection of DNA in capillaries required 5 kV during 3 s. Electrophoresis was carried out at 15 kV and 32°C for about 30 min per sample.

#### Quantitative PCR (q-PCR)

PCR reactions were prepared using 96-wells real-time PCR plates (Eppendorf Hamburg, Germany) and a Mastercycler ep gradient S (Eppendorf Hamburg, Germany). 12.5 μL of Express qPCR Supermix with premixed ROX (Invitrogen, France), 5 μL of DNA extracts with three appropriate dilutions, 200 nM of the forward and reverse primers [23], 50 nM of the TaqMan probe, and water were added to obtain a final volume of 25 μL for all analyses.

An initial incubation of 20 s at 95°C and 40 cycles of denaturation (95°C, 15 s; 60°C, 1 min) were performed. One standard curve was generated at each assay, using 10-fold dilutions in sterilized water (Aguettant Laboratory, Lyon, France) of PCR products from known environmental clones. Clones DF10 and LC103 [29] were used as standards for *Archaea* and *Bacteria*, respectively. One measurement at two or three dilutions was obtained per sample for each primer set.

The amplification efficiency in 16S rRNA standard curves for *Bacteria* and *Archaea* ranged from 86 to 96%. The detection limit was 1.6 × 10^5^ and 8.4 × 10^4^ copies per ml of sludge in average for *Bacteria* and *Archaea* respectively.

### Identification of the dominant communities by 454-pyrosequencing

The identification of dominant bacterial communities from all the reactors at steady-state was obtained by 454-pyrosequencing of the V4–V5 regions of the 16S rRNA gene (Molecular Research Laboratory, TX, USA). The universal primer used (515F-928R) allowed the detection of both *Bacteria* and *Archaea*. Sequence data derived from the sequencing was processed using a proprietary analysis pipeline (Molecular Research Laboratory, TX, USA). Briefly, barcodes and primers were depleted and then sequences shorter than 200 bp or with ambiguous base calls were removed. Sequences were denoised and chimeras removed. Operational taxonomic units (OTUs) were defined after removal of singleton sequences and clustering at 3% of divergence [30]. OTUs were then taxonomically classified using BLASTn against a curated GreenGenes database [31]. 454 pyrosequencing data were deposited to the NCBI Sequence Read Archive under the BioProject ID PRJNA252426.

### Statistical analyses

All statistical analyses were done with the R version 2.11.1 software [32]. Differences in bacterial and archaeal densities over the time and between inocula were determined with an analysis of variance (ANOVA) and a Student Newman Keuls test (SNK) [33].

The CE-SSCP profiles were aligned with the internal standard ROX to correct any change in the electrophoretic motility between runs. The sum of the peak areas were normalized to unity before statistical analyses. The peak areas were determined using a rolling-ball algorithm. The Simpson diversity index calculated from SSCP data (*D*_*SSCP*_) was computed for each fingerprinting profile by the formula *D*_*SSCP*_ *=* - *ln*∑(peak areas)^2^, using the implementation of the R StatFingerprints library [34,35]. The Euclidean distance was used as a measure of the genetic distances between communities. Changes in CE-SSCP genetic structure or in dominant communities as found in pyrosequencing data were displayed using principal component analysis (PCA). The correlations between key reactor functioning parameters and fingerprinting profiles of the bacterial and archaeal communities was investigated using the “envfit” function of the R vegan library [36].

The sequence number of each sample was normalized by the sequence number of the smaller samples found. The Simpson diversity index calculated from sequencing data (*D*_*seq*_) was computed by the formula *D*_*seq*_ *=* - *ln*∑(p_i_)^2^, where p_i_ is the relative abundance of each single sequence (OTU).

## Results

### Anaerobic reactor performances

The performances of the anaerobic reactors were estimated using the following parameters: dry matter (DM) and volatile solids (VS) concentrations, chemical oxygen demand (COD), concentration and composition of biogas, pH and volatile fatty acids (VFA) concentrations. Functional steady states, based on organic matter removal efficiency assessment, were observed after 4 hydraulic retention times (HRT). The performance parameters of the spiked triplicate reactors and the control non-spiked reactor for each inoculum at steady state are presented in Table 1. The relative concentration of each VFA is presented in S4 Table.

**Table 1:** Characteristics of the feeding sludge (FS) and of the outlet sludge (*eco 1*, *2 or 3*) from the mesophilic bioreactors inoculated with the three microbial communities at steady state.

Spiking	Sludge	DM *(g*.*L* ^*-1*^ *)*	VS *(g*.*L* ^*-1*^ *)*	CH_4_ *(%)*	CH_4_ *(mL*.*g*_COD*_^-1^ *)*	COD_tot_ *(g*_*O2*_.*L* ^*-1*^ *)*	COD_DCM_ *(g*_*O2*_.*L* ^*-1*^ *)*	pH	VFA_tot_ *(g*_*eqCOD*_.*L* ^*-1*^ *)*
yes	*FS*	16.3 ± 0.5	12.9 ± 0.2			26.4 ± 1.1		7.0 ± 0.2	3.3 ± 0.4
no	*FS* control	16.5 ± 1.4	11.8 ± 1.0			27.3 ± 4.7		7.0 ± 0.2	3.4 ± 0.5
yes	*eco1* (1)	14.8 ± 0.2	10.0 ± 0.2	46 ± 2	85.2 ± 45.9	24.3 ± 1.1	8.6 ± 0.2	5.7 ± 0.1	6.6 ± 0.2
yes	*eco1* (2)	14.5 ± 0.1	9.8 ± 0.2	48 ± 1	76.5 ± 32.6	23.4 ± 0.3	8.6 ± 0.2	5.6 ± 0.1	6.8 ± 0.1
yes	*eco1* (3)	14.4 ± 0.3	9.7 ± 0.3	40 ± 6	71.0 ± 37.5	24.7 ± 0.8	8.9 ± 0.1	5.8 ± 0.1	6.6 ± 0.3
no	*eco1* control	14,5 ± 0.5	9.6 ± 0.3	45 ± 1	57.8 ± 13.8	22.8 ± 2.6	7.4 ± 0.4	5.9 ± 0.1	7.3 ± 1.1
yes	*eco2* (1)	14.8 ± 0.1	10.0 ± 0.1	46 ± 1	118.6 ± 45.6	24.5 ± 0.9	8.5 ± 0.3	5.5 ± 0.1	6.8 ± 0.1
yes	*eco2* (2)	14.5 ± 0.3	9.7 ± 0.3	63 ± 9	187.0 ± 105.9	24.5 ± 0.7	7.4 ± 1.7	6.4 ± 1.0	5.6 ± 1
yes	*eco2* (3)	13.9 ± 0.6	9.3 ± 0.6	79 ± 9	322.8 ± 114.1	19.6 ± 2.9	4.7 ± 1.0	8.1 ± 0.1	3.3 ± 0.7
no	*eco2* control	14.5 ± 0.5	9.6 ± 0.3	64 ± 3	270.5 ± 107.5	22.4 ± 0.7	4.5 ± 0.1	7.9 ± 0.1	4.1 ± 0.4
yes	*eco3* (1)	14.4 ± 0.5	9.7 ± 0.4	78 ± 1	424.5 ± 67.1	20.7 ± 1.3	2.7 ± 0.5	8.1 ± 0.1	1.3 ± 0.5
yes	*eco3* (2)	14.9 ± 0.2	10.0 ± 0.2	76 ± 2	341.6 ± 95.4	20.5 ± 1.7	4.1 ± 0.2	8.2 ± 0.1	2.7 ± 0.1
yes	*eco3* (3)	14.2 ± 0.4	9.5 ± 0.3	78 ± 1	366.4 ± 92.0	18.7 ± 0.6	2.3 ± 0.4	8.1 ± 0.1	1.0 ± 0.4
no	*eco3* control	13.7 ± 0.5	8.6 ± 0.4	72 ± 3	295.1 ± 62.2	15.5 ± 0.7	1.6 ± 0.1	8.0 ± 0.1	0.6 ± 0.3

COD_tot_ is measured on the total sample and COD_DCM_ on the DCM fraction. COD*: degraded COD, nd: not detectable. The VFA_tot_ is the total concentration of the VFA measured on the DCM fraction. The different parameters were averaged on two HRT at steady state.

CH_4_ productions in the spiked reactors were compared to the non-spiked control reactors. Reactors *eco3* produced more CH_4_ than their negative controls; the *eco1* slightly more and the *eco2* produced about the same. This means that spiking has no influence on methane production. Moreover, the spiking had no impact on the other functional parameters. The influence of the origin of the inoculum on the DM and VS removal of the spiked reactor was also studied. Regardless the inoculum, the dry matter removal efficiency was similar and around 12 ± 2%.

All the biogas produced by the reactor was constituted of CH_4_ and CO_2_. The average methane concentrations and proportions were presented in the Table 1. They differed from one inoculum to another (ANOVA, P<0.001). The lowest methane concentrations and proportions were measured for the reactors *eco1* (72.4 ± 34.3 mL.g_COD degraded_^-1^; 44.6 ± 3.9%) and the highest ones for the *eco3* systems (350.2 ± 91.8 mL.g_COD degraded_^-1^; 76.2 ± 2.7%). For the reactors *eco2*, the measured concentrations were more varying from one reactor to another (with methane concentration varying from 118.6 ± 45.6 to 322.8 ± 114.1 mL.g_COD degraded_^-1^ and proportions from 45.8 ± 1.2% to 79.1 ± 9.0%).

The pH differed also according to the inocula, with a higher pH for *eco3* than for *eco1* and *eco2* (Table 1). The VFA concentration reached (in average) 6.6 ± 0.2 g_eqCOD_.L^-1^ in *eco1* and 1.7 ± 0.9 g_eqCOD_.L^-1^ in *eco3*. This difference of VFA concentration was explained by an accumulation of acetate in *eco1* (S4 Table). The same heterogeneity on *eco2* replicates was found for the pH and VFA as mentioned for CH_4_ concentrations.

Apart from the degradation of DM and VS, which are identical regardless of the reactor, the study of macroscopic operating parameters such as VFA concentration, biogas production and pH, revealed differences depending on the origin of the studied inoculum: The activity of *eco3* can be defined as “methanogenic” (i.e with a COD mass balance near the theoretical one of 350 ml CH_4_.g_COD degraded_^-1^ and no accumulation of VFA), *eco1* as “fermentative” (i.e with a partial methanogenic activity: accumulation of VFA and low CH_4_ production) and *eco2* as “intermediate” (i.e. like *eco3* or *eco1*). Indeed, the *eco1* and *eco3* replicates were homogenous while *eco2* replicates were heterogeneous.

### PAH removals

**Fig 1:**
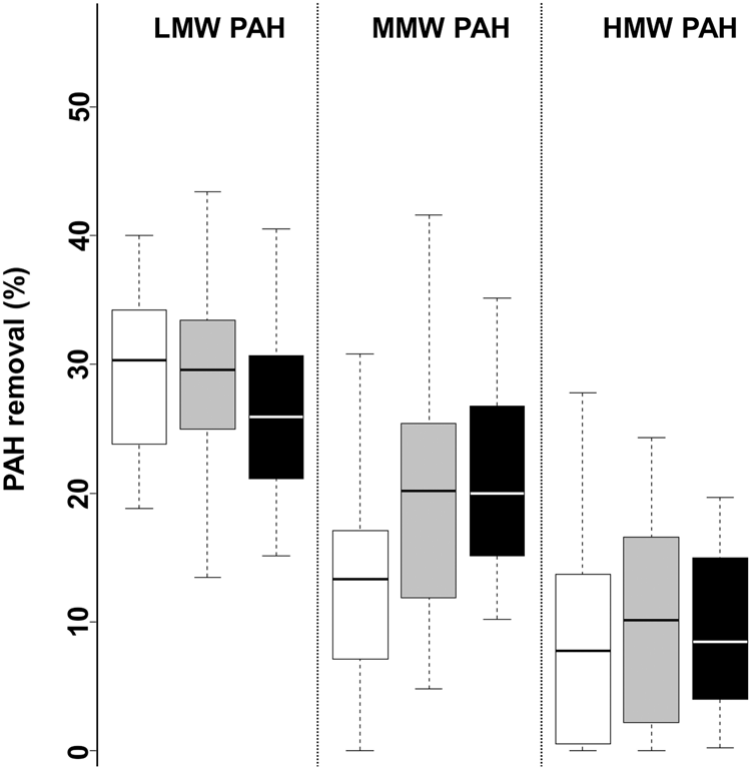
Boxplot of the anaerobic pollutants removal at steady state according to the inocula. The removals for eco1 (white), eco2 (grey) and eco3 (black) were measured at steady state. The LMW-PAH, the MMW-PAH and the HMW-PAH are defined in Table 1. The box shows 75^th^ percentiles (top line), 50^th^ percentile (middle line) and 25^th^ percentile (bottom line).

**Fig 2:**
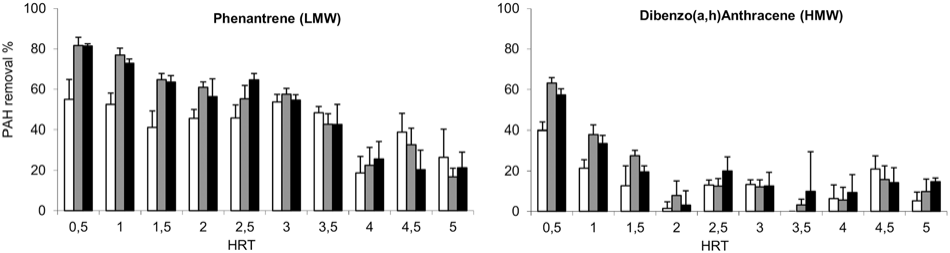
Percentage of PAH removal over the time (expressed as Hydraulic Retention Times) according to the inoculum. Phenanthrene is a representant of the LMW-PAH and Dibenzo(a,h)Anthracene of the HMW-PAH. The performance of the eco1, eco2 and eco3 are respectively in white, grey and black.

The PAH removal efficiencies of each inoculum were studied at steady state (Fig 1) but also over the time (Fig 2).

At steady state and regardless the inoculum (Fig 1), the PAH removal efficiencies were low and decreased from 30% to 10% with the increase of the molecular weight of the PAH considered (ANOVA, P < 0.001). Moreover, PAH removals were similar between the inocula when considering only the low molecular weight (LMW-PAH) (ANOVA, P = 0.059) and the high molecular weight (HMW-PAH) (ANOVA, P = 0.814) categories. For medium molecular weight MMW-PAH category, the soil inoculum displayed the lowest removal (SNK test, P < 0.05).

**Fig 3:**
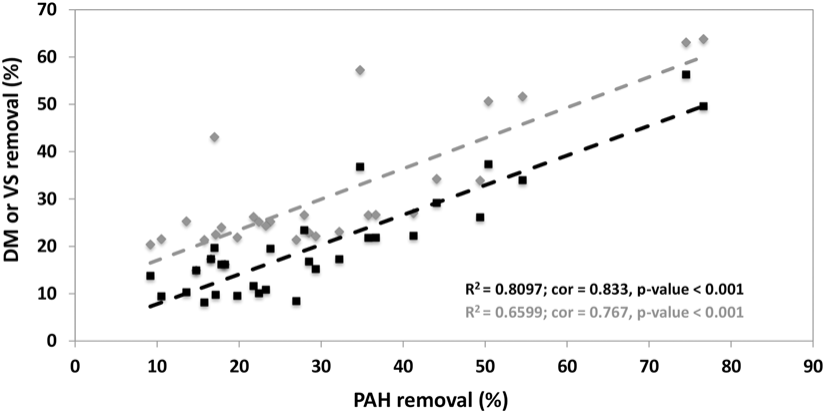
Correlation between DM (black square) or VS (grey triangle) removals and PAH removal.

At the initial state (HRT0), the PAH removal efficiencies differed according to the inoculum (Fig 2). The PAH removal was higher in the *eco2* and *eco3* reactors. The PAH (all molecular weight categories) (S1 Fig), DM and VS (data not shown) concentrations decreased over time and converged at steady state to similar values whatever the inoculum. The DM and VS removals were positively correlated with the PAH removal efficiencies (cor_DM_ = 0.833, cor_VS_ = 0.767) (Fig 3).

At steady state, the PAH removal efficiencies did not depend on the origin of the inoculum. This could have been due to the selection of similar communities by the designed experiment; indeed bioreactors were inoculated with three different inocula but fed with the same spiked and sterilized sludge. The abundance and the diversity of the bacterial and archaeal communities were then checked.

### Changes in abundances of microorganisms

**Fig 4:**
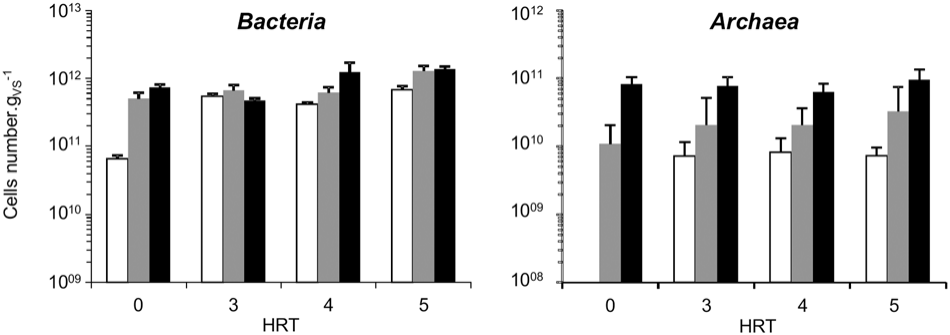
Average enumeration according to the inoculum origin (*eco1* in white, *eco2* in grey, *eco3* in black) of *Bacteria* (left) and *Archaea* (right) by q-PCR. The x axis represents the hydraulic retention time. Standard errors are displayed. HRT4 and HRT 5 represent the steady state based on the reactors performances.

The abundance of microorganisms was examined by q-PCR for each inoculum origin at HRT0, HRT3 and at steady-state (Fig 4). Before any comparison between experimental conditions, we checked if spiking with micropollutants had an effect on abundances: no effect was observed on *Bacteria* abundances (ANOVA; for the soil P = 0.609, the sediment P = 0.521 and the sludge P = 0.844). However, a slight effect was observed for *Archaea* in the reactors inoculated with *eco1* and *eco3* with a lower abundance in the spiking one (ANOVA; P < 0.001).

At steady state, the mean concentration of *Bacteria* per gram of VS was 5.63×10^11^ (± 6.25×10^10^) for *eco1*, 8.99 × 10^11^ (± 1.40 × 10^11^) for *eco2* and slightly higher with 1.31 × 10^12^ (± 7.85 × 10^10^) for *eco3*. For the *Archaea*, the average densities on the triplicated reactors increased according to the inoculum with 7.72 × 10^9^ (± 1.00 × 10^9^) for *eco1*, to 2.77 × 10^10^ (± 8.76 × 10^9^) for *eco2* and 7.80 × 10^10^ (± 8.90 × 10^9^) for *eco3*.

The density of *Bacteria* increased over time (ANOVA, P < 0.001) for *eco1* from 6.46 × 10^10^ (± 8.73 × 10^9^) at HRT0 to 5.63 × 10^11^ (± 6.25 × 10^10^) at HRT5. The same trend is observed for *eco3* (ANOVA, P < 0.001) from 7.39 × 10^11^ (± 7.70 × 10^10^) to 1.31 × 10^12^ (± 7.85 × 10^10^). Conversely, the density of *Bacteria* remained stable over time for *eco2* (ANOVA, P = 0.05) at around 7.56 × 10^11^ (± 8.98 × 10^10^). The dynamics of archaeal concentrations varied depending on the inoculum. The *Archaea* quantity increased in reactors *eco1* from below the detection limit to 7.72 × 10^9^ (± 1.00 × 10^9^). By contrast, the quantity of *Archaea* was similar between initial and final time (SNK, P < 0.05) for the reactors *eco2* with 2.48 × 10^10^ (± 7.43 × 10^9^) and the reactors *eco3* with 7.88 × 10^10^ (± 5.42 × 10^9^). The microbial densities varied over time until reaching a steady state after three HRT.

**Fig 5:**
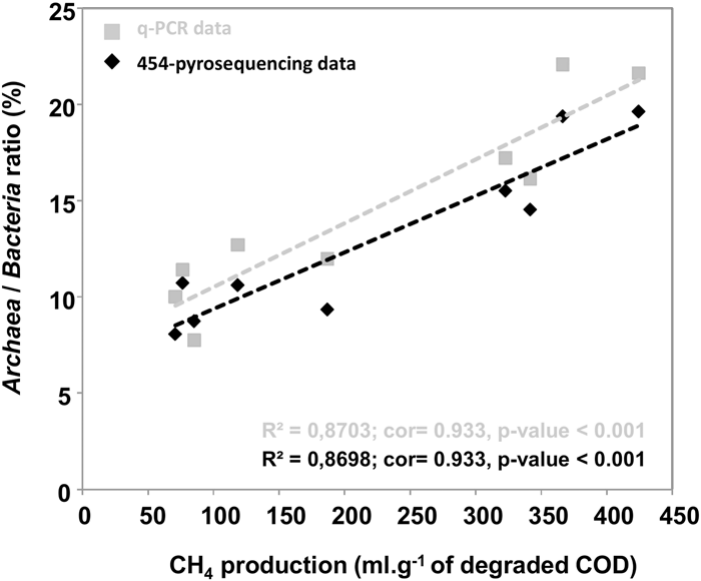
*Archaea* / *Bacteria* ratio depending on the methane production at steady state. The ratio is either calculated with the q-PCR or the 454-pyrosequencing data.

We used these *Bacteria* and *Archaea* densities at steady state to calculate a ratio *Archaea/Bacteria*. A high positive correlation (cor = 0.933, P < 0.001) was found between the methane production and this *Archaea/Bacteria* ratio for all the reactors (Fig 5).

### Microbial community diversity and structure

The structure and the diversity of the microbial communities were examined for each of the twelve reactors over time by using CE-SSCP fingerprints. The *D*_*seq*_ was also calculated from sequencing data at steady state. The number of detected sequences varied from 3364 to 15538 sequences depending on the sample. We then homogenized the sampling effort to the smallest sequence number within the samples [37], even though the Simpson diversity index gives robust results regarding the variation of the sampling size.

According to the CE-SSCP data, the resulting Simpson diversity index (*D*_*SSCP*_) for *Bacteria* varied over the time depending on the inoculum (S5 Table). The *D*_*SSCP*_ decreased from 3.0 to 2.1 for the reactors *eco1*, whereas it increased from 2.7 to 3.2 for the reactors *eco3* and was stable at around 2.5 for the reactors *eco2*. The mean *D*_*SSCP*_ for *Archaea* at steady state in reactors *eco1* was around 2.4. The archaeal *D*_*SSCP*_ decreased from 3.1 to 2.1 in reactors *eco2* and increased from 2.4 to 3.3 in reactors *eco3*. For the *Bacteria*, the comparison at steady state between *D*_*SSCP*_ and *D*_*seq*_ showed no significant differences (T-test, p-value>0.05). The diversity of bacterial and archaeal communities of reactors *eco3* was higher than for the other reactors at steady-state. Moreover, each inoculum still retained its own diversity albeit operating conditions were maintained similar for all the bioreactors for 100 days.

**Fig 6:**
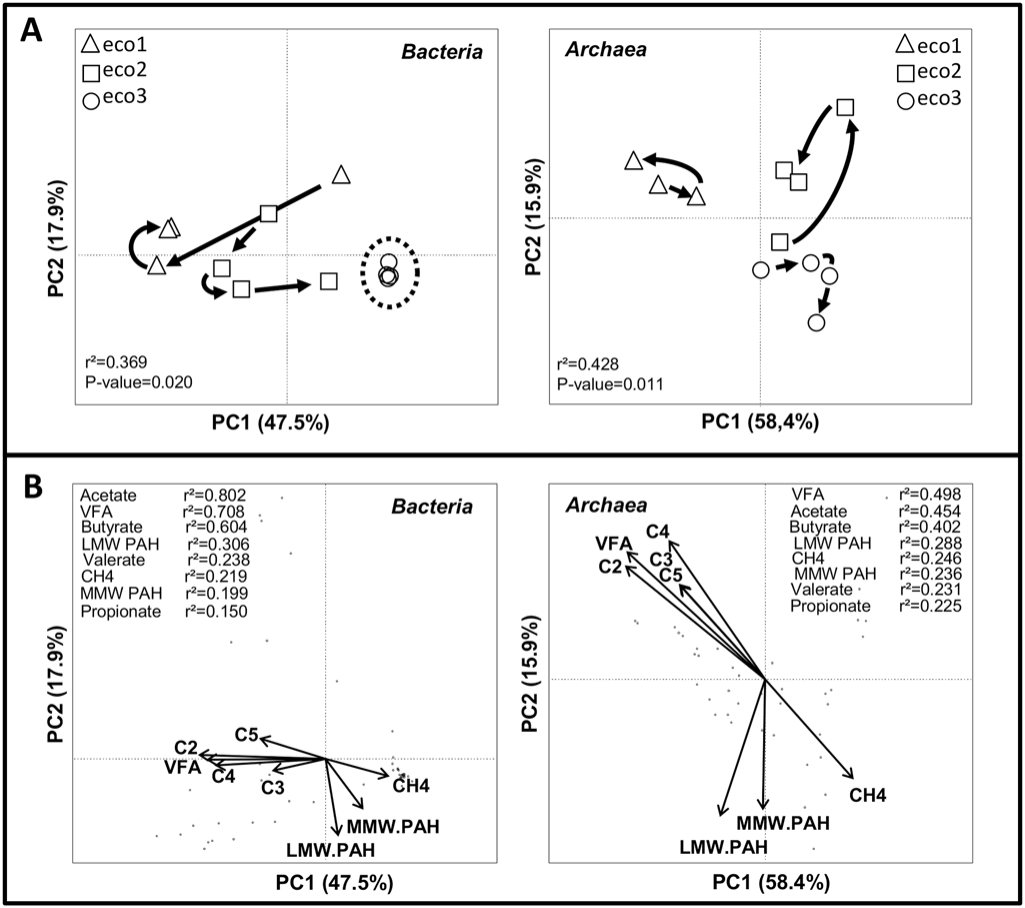
**(A)** Influence of the inoculum origin (*eco1*, *eco*2, *eco3*) and time (HRT0, HRT3, HRT4 and HRT5) on the difference in genetic structure of bacterial (on the left) and archaeal (on the right) communities through a PCA. The percentage variation explained by each principal component and the principal component scores of the sample are plotted on their respective axes. Changes over time are graphically represented by arrows.**(B)** Principal Component Analysis (PCA) biplot of bacterial (on the left) and archaeal (on the right) communities. PCA displayed 65.4% of variance for bacterial CE-SSCP fingerprints and 74.3% of variance for archaeal CE-SSCP fingerprints. Only significant correlations with operational characteristics were presented as arrows.

When combining molecular microbial ecology measurements with data about operation and functioning, the resulting dataset is highly multivariable. Statistical multivariate analyses, such as Principal Component Analysis (PCA) is then needed to aggregate data and to identify putative causal factors of changes in microbial communities among the parameters studied [38,39]. Fig 6 displays Principal Component Analysis (PCA) ordinations, indicating shifts in community structures according to the inoculum source and to the sampling time during the experiment. The PCA displayed 65.4% of the genetic differences on the two first principal components when comparing bacterial CE-SSCP fingerprints and 74.3% of the genetic differences when comparing archaeal CE-SSCP fingerprints. The design of the common garden experiment (with three replicated communities from three different inocula fed with the same spiked sludge) allowed deciphering the role of the inoculum source on the pollutants removal. Here, both bacterial (P < 0.001, r^2^ = 0.245) and archaeal (P < 0.001, r^2^ = 0.351) community structures could be discriminated according to the inoculum source. The clustering of the bacterial (P = 0.02, r^2^ = 0.369) and the archaeal (P = 0.01, r^2^ = 0.428) communities according to the inoculum source were improved when the duration of the experiment was also considered (Fig 6A).

The global functioning of reactors was shown to be different according to the inoculum source (Table 1). Significant correlations between some parameters of reactors functioning (Fig 6B) and community structures (P < 0.02) are represented as arrows. The length and orientation of arrows indicated the proportional importance and direction of correlated factors. The VFA concentration was the most highly correlated parameter of reactors functioning with changes in the genetic structure of *Bacteria* (r^2^ = 0.708) and *Archaea* (r^2^ = 0.498). The arrows indicating VFA accumulations were directed toward mostly CE-SSCP fingerprints from *eco1* and *eco2* samples. By contrast, the arrows for CH_4_ production were directed toward CE-SSCP fingerprints from *eco3* samples. Changes in CE-SSCP fingerprints in relation with LMW-PAH and MMW-PAH removals were consistent for bacterial and archaeal community structures, but theses variations were independent to the other parameters of reactor functioning, like VFA or CH_4_.

**Fig 7:**
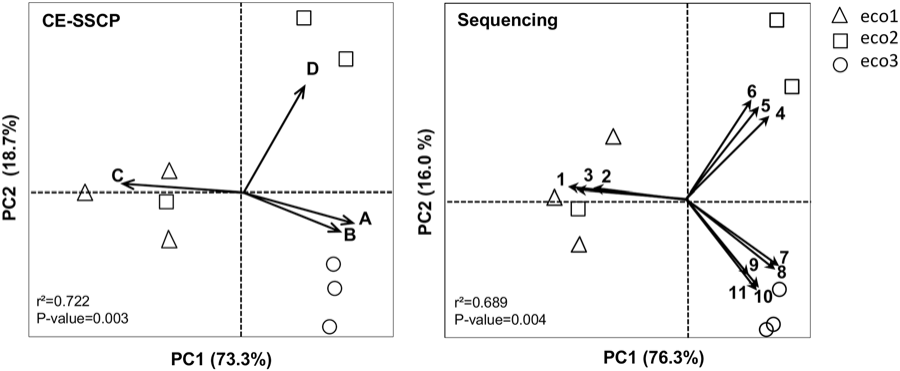
Principal Component Analysis (PCA) biplot at steady state of microbial communities (*eco1*, *eco2*, *eco3*) obtained with the CE-SSCP data (on the left) and with the sequencing data (on the right). PCA displayed 92% and 92.3% of variance for microbial fingerprints and sequencing respectively. Most discriminant CE-SSCP peaks (on the left) and most discriminant species (on the right) were highlighted as arrows that were directed according to their explanatory outputs. 1, 2, 3: *Clostridium sp*. (88%), 4: *Parabacteroides sp*. (87%), 5: *Dysgonomas sp*. (90%), 6: *Parabacteroides sp*. (86%), 7: *Clostridium sp* (93%), 8: *Anaerobaculum mobile* (99%), 9: *Thiohalomonas sp*. (82%), 10: *Op9* (99%), 11: *Pseudomonas stutzeri* (99%).

A strong agreement between CE-SSCP community structures and 454-pyrosequencing data was observed for all the reactors at steady state (Fig 7) and underlined the similar explanatory power of both technologies. Whatever the methods, the samples were indeed clustered according to the origin of the inoculum. The major phyla that characterized the reactors were the *Firmicutes*, *Bacteroides*, *Synergitetes* and *Proteobacteria* (S1 Fig). The relative abundance of each phylum varied according to the inoculum origin. All the reactors *eco3* were characterized by two discriminant peaks (peak A and peak B) corresponding, according to the sequencing results, to several bacteria affiliated to the phyla *Firmicutes*, *Synergitetes*, *Proteobacteria* and *OP9*. The genus *Synthrophomonas* was also mainly associated with the reactors *eco3* (cor = 0.860, P = 0.003). All the reactors *eco1* and one replicate of the reactors *eco2* were characterized by the presence of one discriminant peak (peak C) corresponding, according to the sequencing results, to a bacterium affiliated to the genus *Clostridium*. Two replica of the reactors *eco2* were characterized by one discriminant peak (peak D) corresponding to three bacterial species affiliated, according to the sequencing results, to the phylum *Bacteroides* (*Parabacteroides* and *Dysgonomonas* genera). The cumulated abundances of sequences affiliated to the *Clostridium* genus, generally associated with a fermentative metabolism, were correlated with the VFA concentration (cor = 0.851, P = 0.003).

The analysis of archaeal communities shows that *Methanobacterium* genus was dominant and represented 54% to 100% of all archaeal sequences. The other *Archaea* were affiliated to *Methanosarcina* genus. No difference was observed between inoculum.

A similar explanatory power of the fingerprinting technique and the 454-pyrosequencing technology was observed at the community scale, despite the much more detailed information achieved by sequencing data. More interestingly, when focusing on the methanogenic activity and the archaeal abundance, we also found a strong and positive correlation between the methane production and the abundance of *Archaea*, either measured by quantitative PCR or 454-pyrosequencing (Fig 5; cor = 0.933, P < 0.001).

## Discussion

This study aimed at unraveling the role of the ecosystem composition and functioning on the biodegradation of persistent organic pollutants during anaerobic digestion. For the first time, series of reactors were inoculated with three different extracted microbial communities, and fed with the same sterilized, PAH spiked sludge for 100 days, with the same matter and pollutant loads. The presence of pollutants had no measurable effect on the abundance of bacterial communities, when compared to the control reactors where no pollutants were added. It suggests a non inhibitory effect of pollutant on the microbial communities.

During the three first hydraulic retention times (HRT) of continuous cultivation, the three inoculated microbial communities adapted gradually to the same feeding sludge and operating conditions. The variations in relative abundances of the communities, in density of *Bacteria* and *Archaea*, and in functioning parameters were monitored. During this adaptation time, we observed a correlated decrease of DM (or VS) and PAH removals. After four HRT, the removal of organic matter (e.g. DM, VS) and the production and composition of the biogas were stable for all the reactors. Similarly, the PAH removal efficiency and the structure and density of the microbial communities became constant in each reactor. As expected, a functional steady state was achieved after four HRT [9]. Interestingly, this functional steady state varied according to the inoculum. Indeed, the statistical analysis of bacterial and archaeal genetic structures showed that the initial differences due to the origins of the inocula were maintained and were even more distinguishable at steady state (Fig 6). The experimental setup allowed us to keep three various microbial communities expressing various methane production rates. We could indeed distinguish between three different behaviors: qualified here as “fermentative”, “methanogenic” and “intermediate” behavior (Table 1). The reactors *eco1* had a “fermentative” activity with a low methane production rate (less than 100 mL.g _degraded COD_^-1^). They presented an accumulation of VFA, especially acetate, a low pH below 6, one dominant bacterial population (peak C) (Fig 6) affiliated to the *Clostridium* genus and a dominant archaeal hydrogenotrophic genus (*Methanobacterium*) versus low dominant acetoclastic genus (*Methanosarcina*). At the opposite, the reactors *eco3* had a “methanogenic” functioning (anti-correlation of VFA concentration and CH_4_ proportion). They were characterized by low VFA concentrations, an alkaline pH, a complete COD mass balance (near the theoretical mass balance 350 mL.g_COD degraded_^-1^) with two dominant bacterial populations in CE-SSCP (peak A and peak B), corresponding to *Firmicutes*, *Synergitetes* and *Proteobacteria* using 454-pyrosequencing (Fig 7). All these taxa were typically found in methanogenic and/or anaerobic ecosystems [40–42]. The reactors *eco2* behaved differently: one assay (*eco2-1*) presented a “fermentative” activity and their microbial species at steady state were similar to those of *eco1*, one assay (*eco2-3*) behaved like a “methanogenic” reactor with microbial species similar to those of *eco3* and the last assay (*eco2-2*) had an “intermediate” functioning. The various microbial adaptation processes during the three first HRT could explain these differences between the replicates of *eco2*. Indeed, during this period, the microbial communities from one inoculum can diverge and the abundances of major phyla can vary. In our case, at steady state, the repartition of major phyla of *eco2-1* was similar to the one in *eco1* characterized by a majority of *Firmicutes*. *Eco2-3* behaved like *eco3* with a higher diversity of phyla and the *Bacteroides* was the major phylum in *eco2-2*. It suggested also that the inoculum *eco2* contained all these phyla at the beginning of the experiment and that the operating conditions could favor one or all of these phyla.

It is interesting to note that the methane production rate was related to the density of *Archaea*, as shown by different molecular techniques (Fig 5). This good agreement between activity and quantitative PCR or 454-pyrosequencing data was already recorded in other systems [43]. The number of *Archaea* is thus relevant to be used as a functional bioindicator.

The fact that in our study all the reactors were fed with the same sterile sludge, under the same experimental conditions, allows us to state clearly that differences of matter degradation (CH_4_, VFA) can only be attributed to differences in structure and relative abundance of microbial communities. The correlations between *Archaea/Bacteria* ratio and CH_4_ production, and between the bacterial genus *Clostridium* and the VFA concentrations, and the anti-correlation between *Synthrophomonas* genus and VFA concentrations, suggested that the microbial structure and the abundance of *Archaea* and of some archaeal/bacterial genus drive each ecosystem to a “fermentative” or “methanogenic” activity. The lower efficiency to produce methane of microorganisms from *eco1* and *eco2* can be explained by the nature of the inocula. Indeed, microorganisms from soil or sediment, environments much more subjected to aerobic conditions, are less adapted to strictly anaerobic conditions than those readily extracted from anaerobic sludge [44].

Therefore, if the reactors could be distinguished according to their inoculum origin and their global functioning, the PAH removal and the dry matter removal were similar at steady state. The structure of communities and the reactor functioning were more driven by the global operating conditions than by the presence of pollutants. Moreover, no correlation was found between the communities evolution and the PAH degradation. This could suggest that either the three communities expressing various methane potential are able to simultaneously degrade PAH, suggesting a non-specific PAH degrading community, or the method used to depict the ecosystem only targets the main species and is unable to trap the specific and minor PAH degrading community.

Up to now, little is known on PAH degradation (pathway and community) under anaerobic conditions and no specific genes of PAH degradation were identified under methanogenic condition. Trably et al. [10] found similar removals with reactors inoculated with a non-adapted to PAH sludge. In our study, at steady state, the levels of PAH removal efficiencies were around 30% for LMW-PAH, 20% for the MMW-PAH and 10% for HMW-PAH. The removal rates in μg/L/day provide the same tendency with low values: 1.18 μg/L/day for LMW-PAH, 1.91 μg/L/day for the MMW-PAH and 1.00 μg/L/day for HMW-PAH. These values were however in the lower range of measurements for PAH removals recorded on mesophilic anaerobic reactors [9], on reactors fed with adapted to PAH sludge [10], or on measurements obtained from batch experiments [14]. In the literature cited, the feeding with non-sterilized sludge served also as a source of active biomass. The actual performance thus represented the combination of the performance of microorganisms coming from feeding and others coming from the inoculum itself. In our study, the concentration of active biomass derived only from the growth, the adaptation and the maintenance of the extracted and re-inoculated biomass [23], which can explain the low levels of PAH removals.

Similarly to PAH removal, organic matter degradation levels at steady state were in the lower range compared to the degradation potential previously reported for mesophilic methanogenic sludge degradation [9]. The gains and losses of diversity indexes over the time of the experiment were relatively tiny but more strikingly, an overall low diversity index was observed as compared to the literature for similar ecosystems [42,45]. This low diversity may affect the performance of general functions such as DM or VS degradation.

The DM or VS and PAH removal efficiencies were positively correlated (Fig 3) which explains their similar decrease over the time. This correlation was already found between these two parameters [9,10]. This result strengthens the idea that the PAH removal is intimately linked to the DM degradation suggesting co-metabolism with one step of the anaerobic digestion. Some studies have tried to define which step of digestion is responsible for this co-metabolism [16,46,47]. It appears that the presence of *Archaea* [16,46] or coupling between the degradation of PAH and methanogenesis via the conversion of by-product (hydrogen and acetate) into methane [15] is required for the degradation of PAH. The implication of *Archaea* in the metabolism of these pollutants should involve syntrophic process between fermentative and methanogenic communities. Indeed, the partial inhibition of the PAH degradation in the presence of bromoethanesulfonate which is recognized to hinder the activity of *Archaea* and also of other microorganisms of the upper anaerobic pathway [48], suggests that microbial actors of methanogenesis could not be those directly involved in the degradation of PAH. Recently, in batch reactor, Cea-Barcia *et al*. [47] showed that PAH removal was coupled to the first steps of anaerobic digestion as acidogenesis and acetogenesis. In our study, regardless the amount and the composition of biogas produced, all reactors have the same PAH removal efficiencies. Clearly, the micropollutants removals did not rely on the global functioning either “fermentative” or “methanogenic” during anaerobic digestion but were correlated positively to the DM removal. These results support the hypothesis that co-metabolism implied in the degradation of PAH may be realized at earlier stages in the production of methane, i.e. the first stages of anaerobic digestion.

*Archaea* present in our samples did not match with the genera *Methanosaeta* and *Methanoculleus*, playing an important role in the metabolism of LMW-PAHs as suggested by Berdugo-Clavijo [19]. But other bacterial genera like *Firmicutes* [16] and *Clostridium* [18] and members of another archaeal family *Methanobacteriaceae* [14], already shown to be involved in the degradation of PAHs under enriched culture conditions, are also present in our reactors. These species may play a role in our systems but their abundances can’t explain the PAH removal. In our systems, unlike the enriched systems described in the literature, PAHs were not the only available carbon source, this can explain the difficulty to link bacterial or archaeal abundances to PAH removal. However, under such complex conditions, PAH degradation seems to be related to the early stages of anaerobic digestion.

## Conclusions

The study on the influence of complex anaerobic microbial communities with contrasting pollution histories on organic micropollutant removals, under the same organo-mineral condition, is a challenging topic. The original experimental strategy used in this study, based on microbial communities extraction re-introduced in a single sterilized and characterized feeding sludge can meet this research objective.

The applied strategy has preserved the genetic difference of microbial communities based on the origin of the inocula. In steady state, this differentiation according to the origin of the inocula was even more distinguishable.

The study of PAH removal, production of biogas and other functioning parameters on the one hand and the characterization of the microbial communities on the other hand, allowed us to demonstrate the independence of the microbial communities genetic structures in the removal of pollutants. This suggests the presence of a non-specific PAH degrading community or a specific one but with a low abundance that our method cannot depict. The differences in genetic structures were however strongly correlated to matter degrading efficiencies either through “fermentative” or “methanogenic” ways. Because the pollutant removal is observed under each “fermentative” and “methanogenic” conditions, it supports strongly the hypothesis that the first steps of the complex anaerobic pathway are implied in this removal maybe by co-metabolism phenomena.

## Supporting Information

S1 Fig PAH removal over the time according to the inoculum. The percentage of removal is indicated in the Y-axis and the number of hydraulic retention times is indicated in the X-axis. The performances of eco1, eco2 and eco3 reactors are respectively in white, light grey and dark grey.
(TIFF)

S2 Fig Repartition of major phyla in all reactors. (TIFF)

S1 Table Physicochemical characteristics of PAHs. nC: number of carbon ring, LMW: Low Molecular Weight, MMW: Medium Molecular Weight, HMW: Hight Molecular Weight.
(TIFF)

S2 Table Primers used during CE-SSCP PCR and q-PCR. (TIFF)

S3 Table PCR conditions for amplification of bacterial an archaeal sequences. (TIFF)

S4 Table Proportion of each VFA measured in feeding sludge (FS) and in outlet sludge reactor (*eco 1*, *2*, or *3*) at steady state. The different parameters were averaged on two HRT at steady state. nd: not detectable.
(TIFF)

S5 Table Simpson diversity index of bacterial and archaeal communities at initial and final HRT. D (SSCP) and D (Seq) are the indexes calculated from CE-SSCP and sequencing data respectively.
(TIFF)
