# Supplementary figures and images for: Similar PAH Fate in Anaerobic Digesters Inoculated with Three Microbial Communities Accumulating Either Volatile Fatty Acids or Methane
Source: PLoS One. 2015 Apr 15;10(4):e0125552. doi: 10.1371/journal.pone.0125552 (PMC4398385; doi:10.1371/journal.pone.0125552)

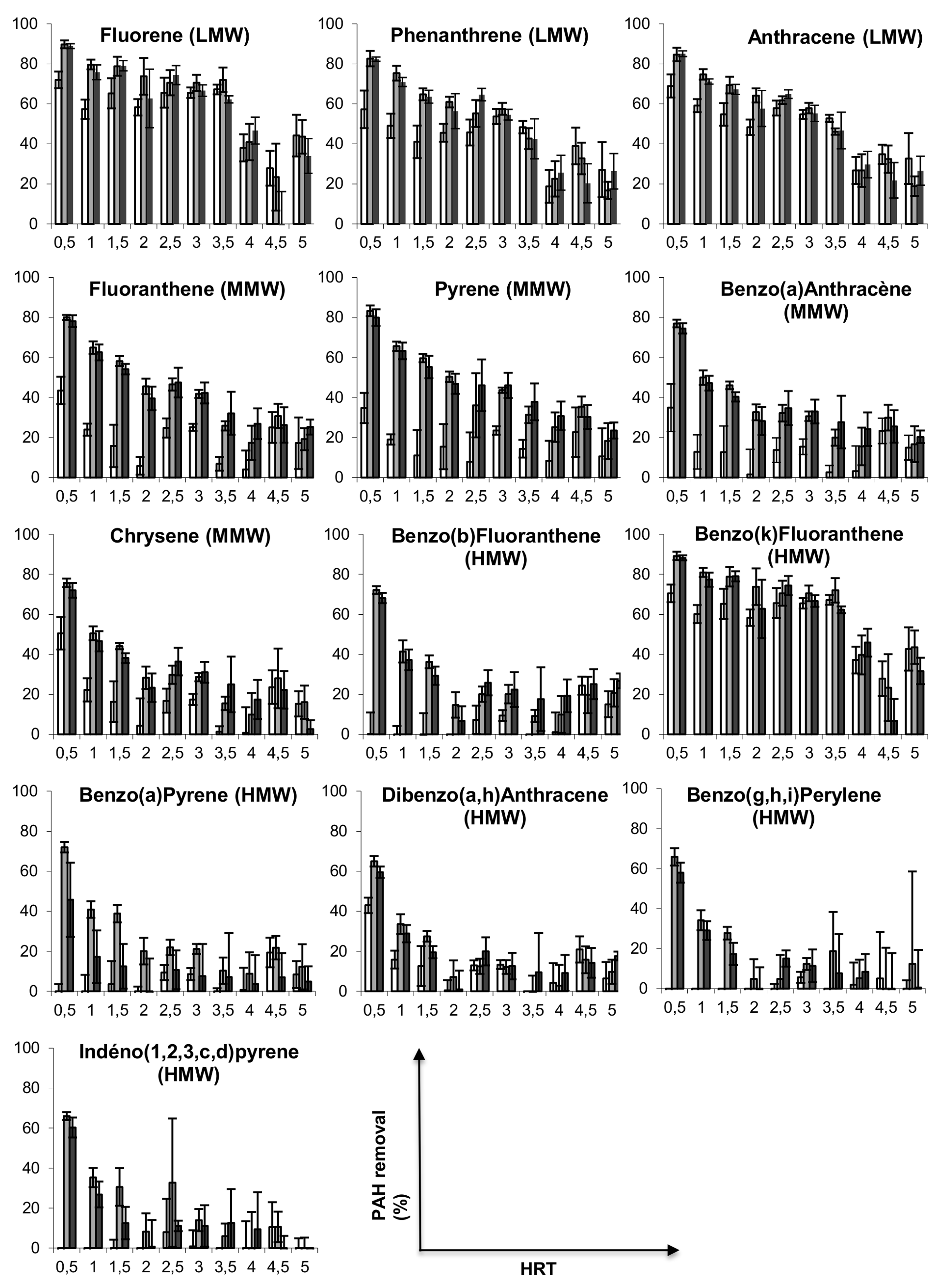

Supplement: S1 Fig — The percentage of removal is indicated in the Y-axis and the number of hydraulic retention times is indicated in the X-axis. The performances of eco1, eco2 and eco3 reactors are respectively in white, light grey and dark grey. (TIFF) [file pone.0125552.s001.tiff]

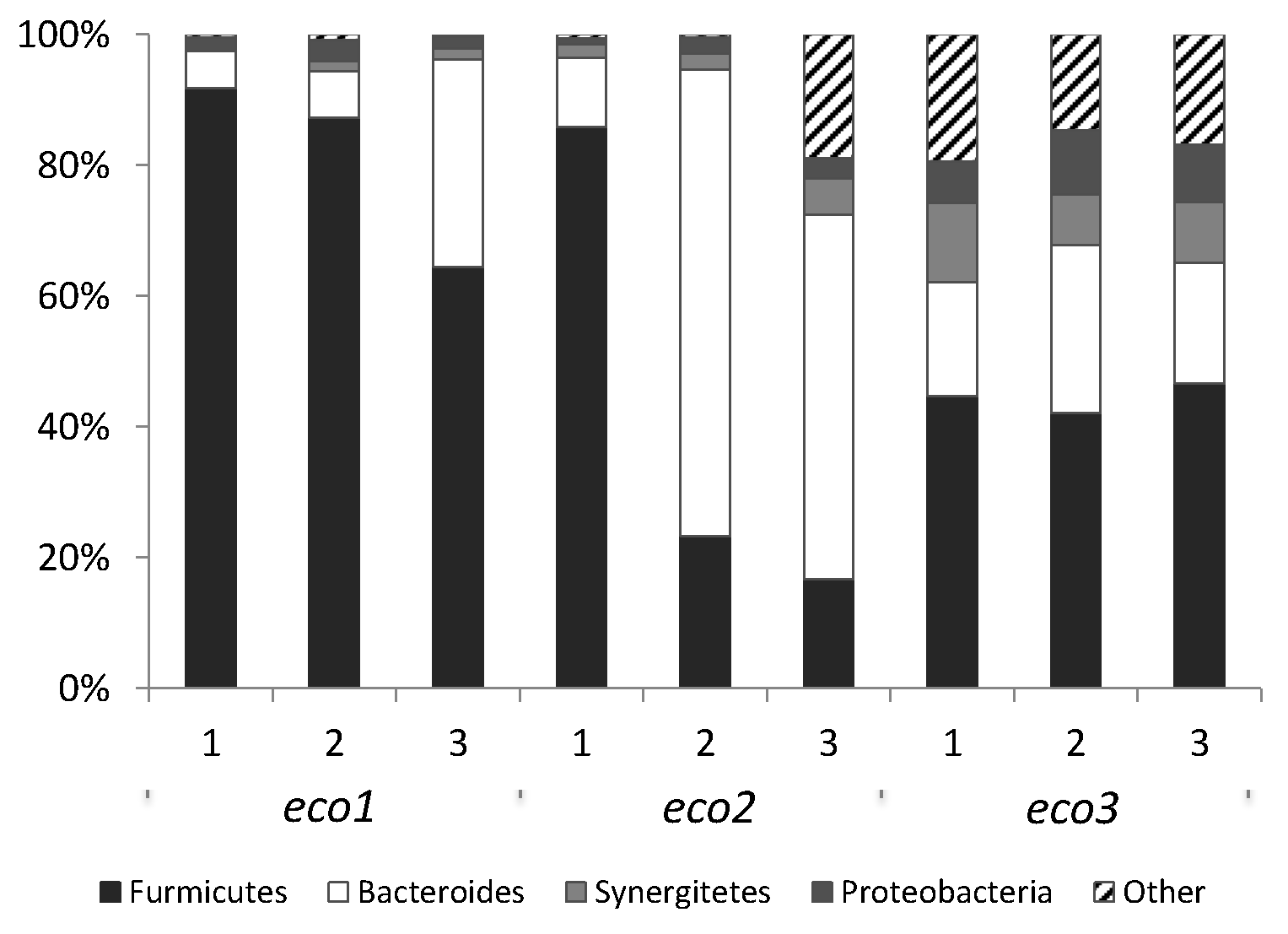

Supplement: S2 Fig — (TIFF) [file pone.0125552.s002.tiff]

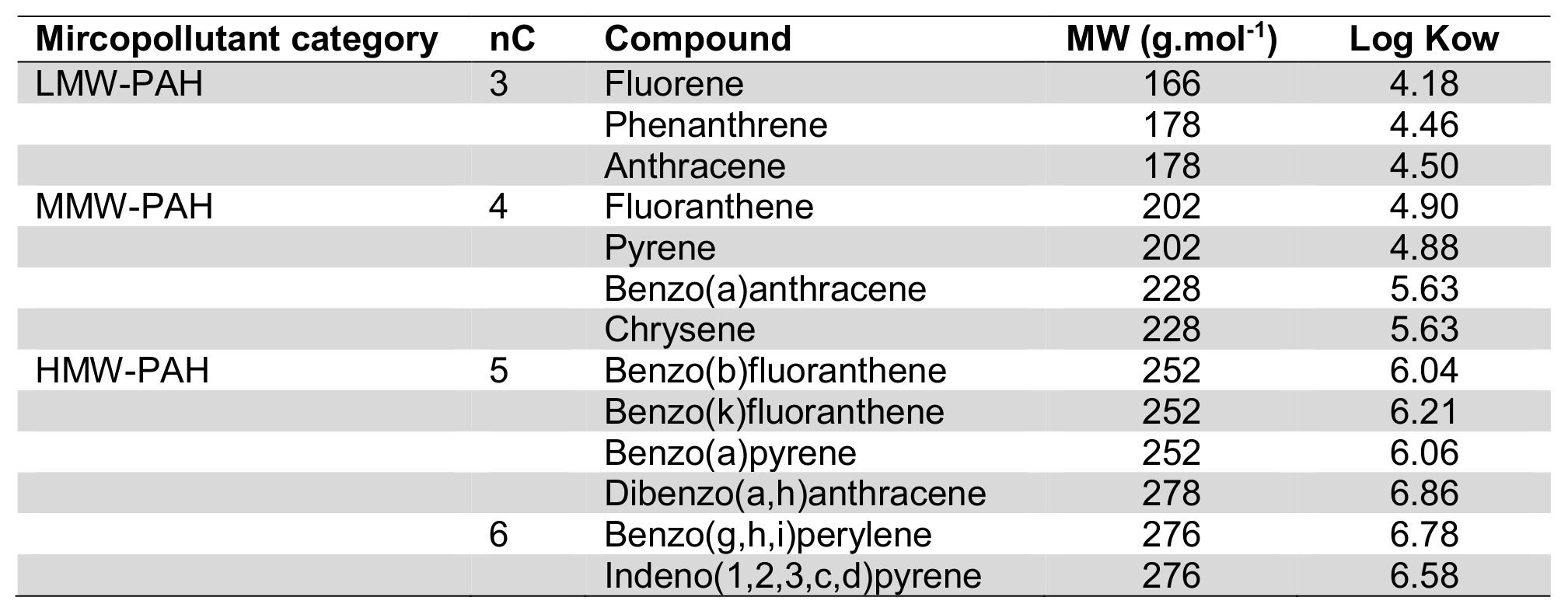

Supplement: S1 Table — nC: number of carbon ring, LMW: Low Molecular Weight, MMW: Medium Molecular Weight, HMW: Hight Molecular Weight. (TIFF) [file pone.0125552.s003.tiff]

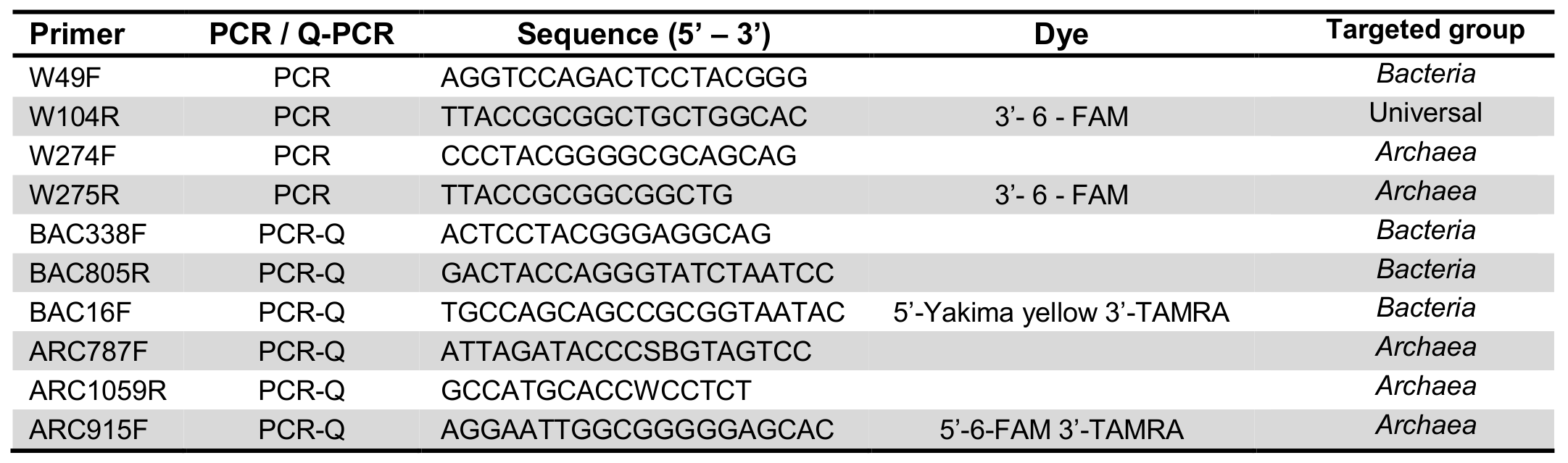

Supplement: S2 Table — (TIFF) [file pone.0125552.s004.tiff]

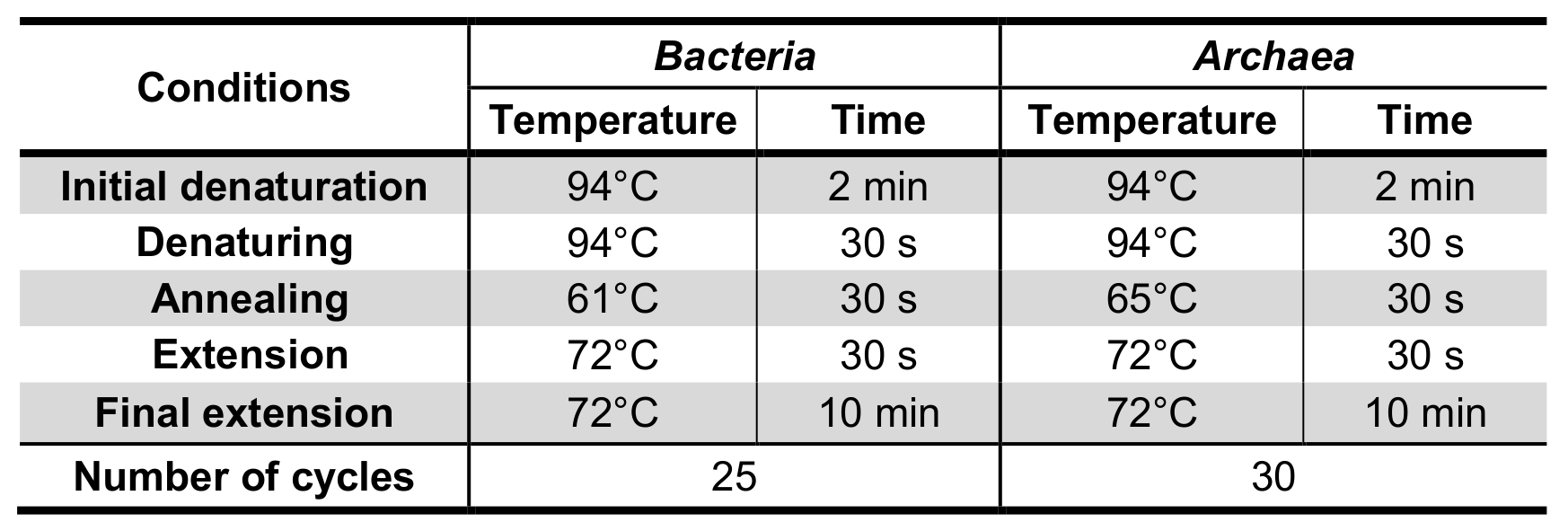

Supplement: S3 Table — (TIFF) [file pone.0125552.s005.tiff]

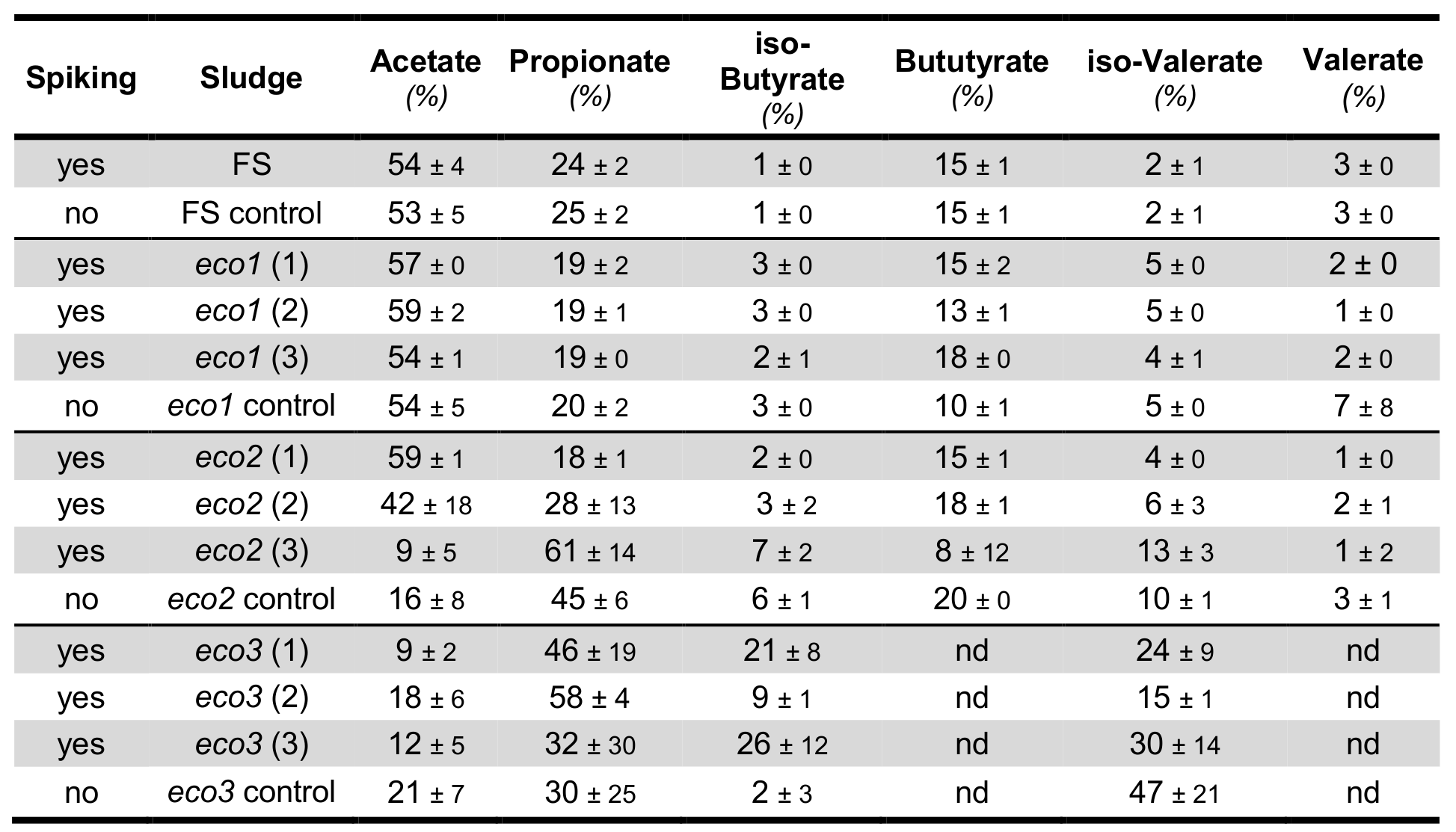

Supplement: S4 Table — The different parameters were averaged on two HRT at steady state. nd: not detectable. (TIFF) [file pone.0125552.s006.tiff]

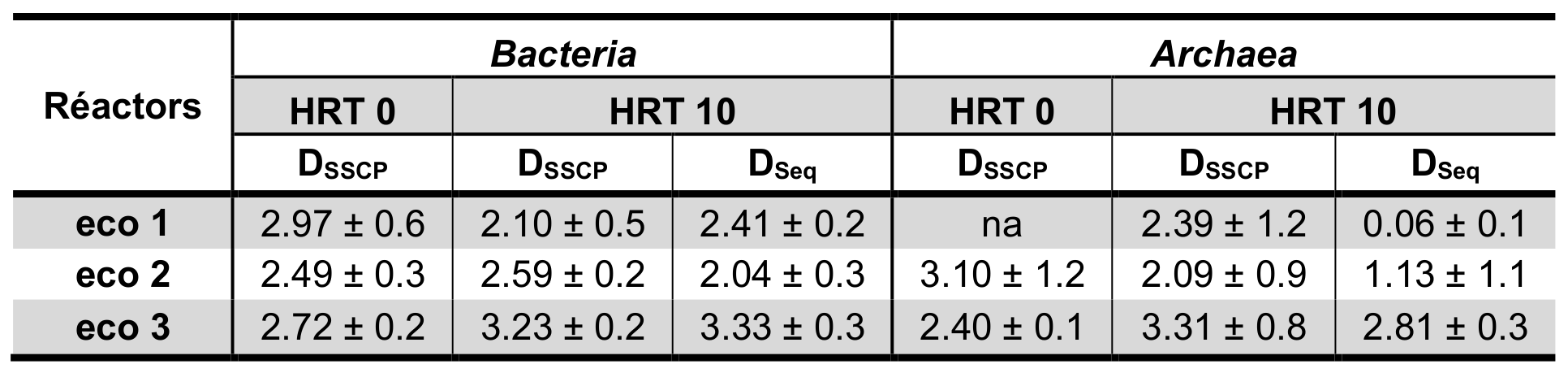

Supplement: S5 Table — D (SSCP) and D (Seq) are the indexes calculated from CE-SSCP and sequencing data respectively. (TIFF) [file pone.0125552.s007.tiff]
